# Supplementary material for: Isotype-aware inference of B cell clonal lineage trees from single-cell sequencing data
Source: Cell Genom. 2024 Aug 28;4(9):100637. doi: 10.1016/j.xgen.2024.100637 (PMC11480863; doi:10.1016/j.xgen.2024.100637)
Supplement: Document S1. Figures S1–S12 and Tables S1 and S2 [file mmc1.pdf]

Cell Genomics, Volume *4*

Supplemental information

Isotype-aware inference of B cell clonal lineage trees from single-cell sequencing data

Leah L. Weber, Derek Reiman, Mrinmoy S. Roddur, Yuanyuan Qi, Mohammed El-Kebir, and Aly A. Khan

Supplemental information — Isotype-aware Inference of B cell Clonal Lineage Trees from Single-cell Sequencing Data

Leah L. Weber[1], Derek Reiman[2], Mrinmoy S. Roddur[1], Yuanyuan Qi[1], Mohammed El-Kebir[1,5,†], and Aly A. Khan[2,3,4,†]

[1]Department of Computer Science, University of Illinois at Urbana-Champaign, IL 61801, USA
[2]Toyota Technological Institute at Chicago, Chicago, IL 60637, USA
[3]Department of Pathology, University of Chicago, Chicago, IL 60637, USA
[4]Chan Zuckerberg Biohub Chicago, Chicago, IL 60642, USA
[5]Lead contact
[†]Correspondence: melkebir@illinois.edu,aakhan@uchicago.edu

Supplemental Figures and Tables

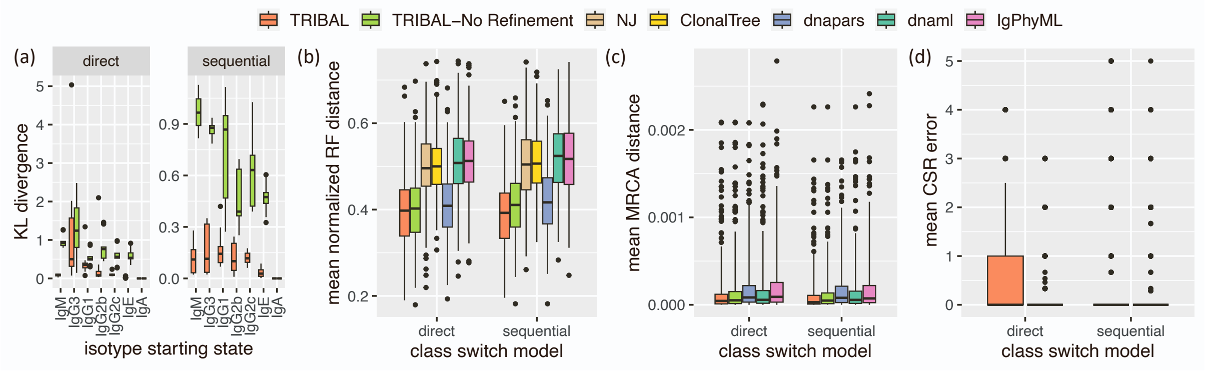

Figure S1. **Simulations results for $k = 75$ clonotypes and $n = 65$ cells per clonotype**, Related to Figure 3.

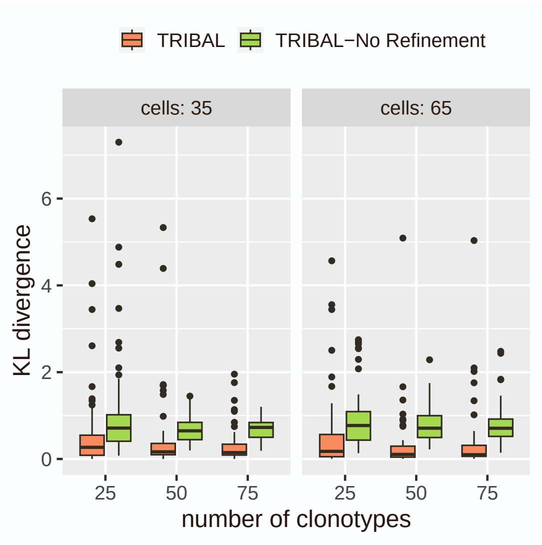

(A) direct CSR model

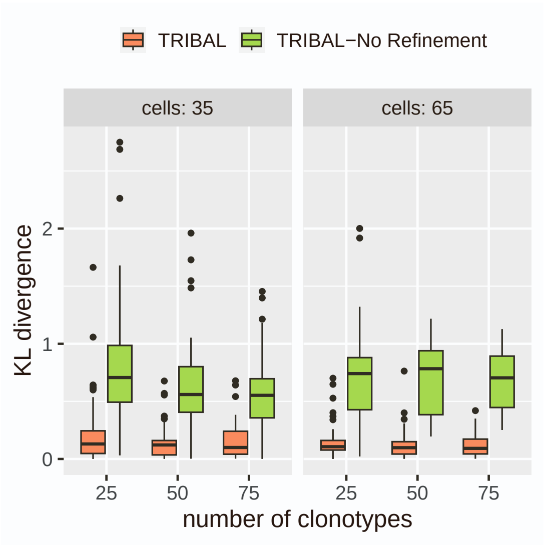

(B) sequential CSR model

Figure S2. KL divergence from ground truth isotype transition probabilities aggregated over all isotype starting states, except IgA, by isotype starting state with varying the number k clonotypes, the number n of cells and CSR model, Related to Figure 3.

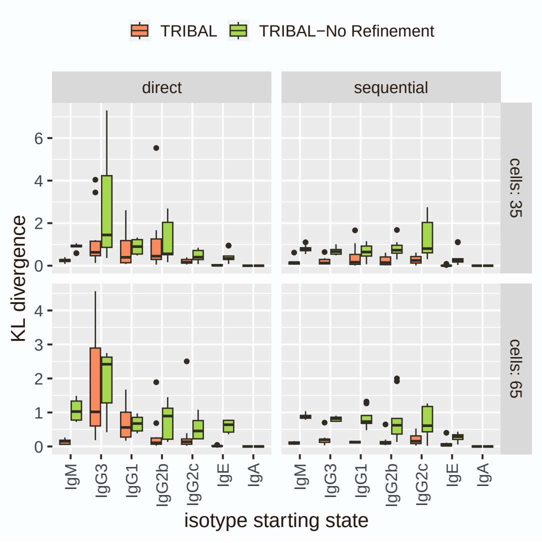

(A) $k = 25$ clonotypes

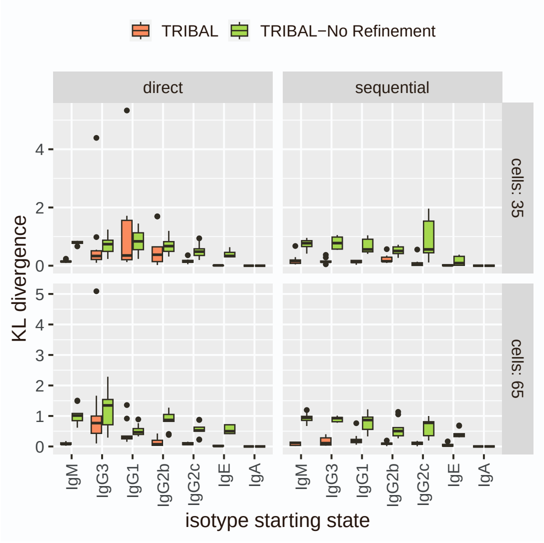

(B) $k = 50$ clonotypes

Figure S3. KL divergence from ground truth isotype transition probabilities by isotype starting state for $k \in \{25, 50\}$ clonotypes, Related to Figure 3.

dataset	clonotypes k	total cells n	median cells per clonotype	max cells per clonotype	median distinct isotypes per clonotype
NP-KLH-1	167	1776	7	89	3
NP-KLH-2a	70	537	6	32	2
NP-KLH-2b	58	357	5	21	2

Table S1. Summary of NP-KLH mouse scRNA-seq datasets, Related to Figure 4.

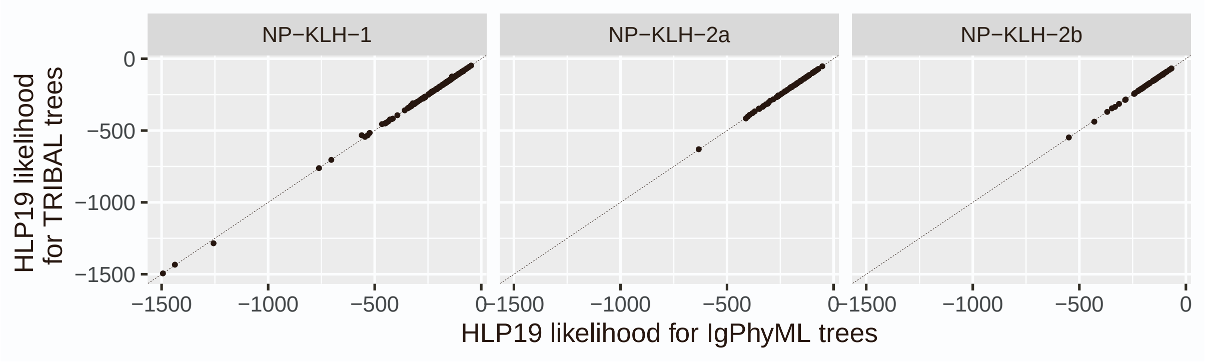

Figure S4. Comparison of HLP19 likelihood computed for IgPhyML and TRIBAL inferred B cell lineage trees for NP-KLH datasets, Related to Figure 4.

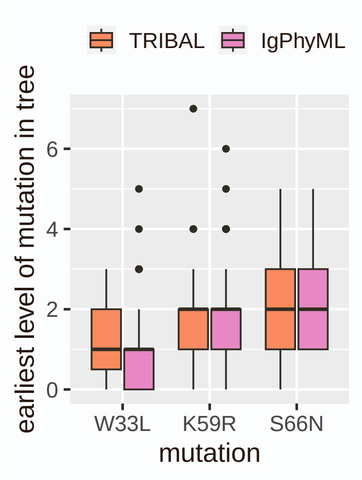

Figure S5. Earliest observed level of mutation in a B cell lineage tree, Related to Figure 4. Level 0 represents the MRCA.

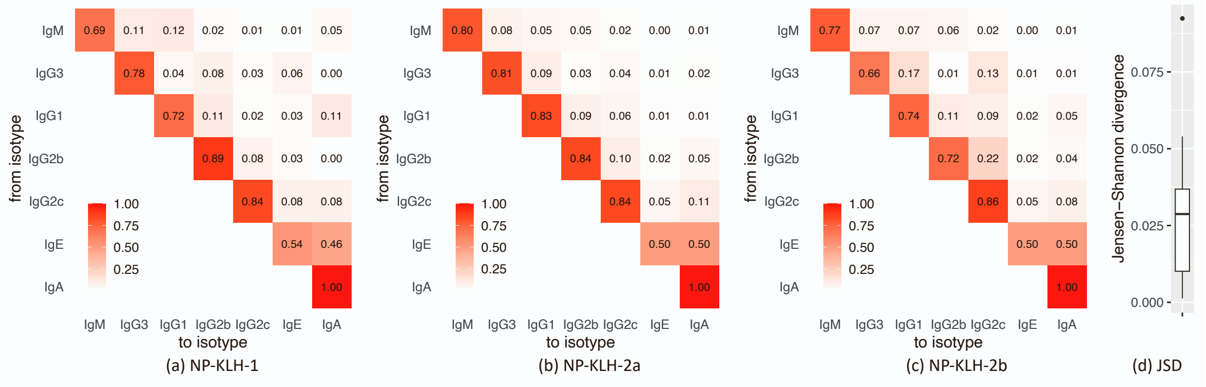

Figure S6. TRIBAL inferred isotype transition probabilities for NP-KLH, Related to Figure 4. (a) Isotype transition probabilities for NP-KLH-1. (b) Isotype transition probabilities for NP-KLH-2a. (c) Isotype transition probabilities for NP-KLH-2b. (d) The distribution of Jensen-Shannon divergence (JSD) for pairwise comparisons of rows of the inferred isotype transition probabilty matrices for IgM through Ig2c. IgE was excluded from comparison due to a lack of observed B cells within each dataset to yield informative estimates. IgA is excluded as the inference of this row is trivial.

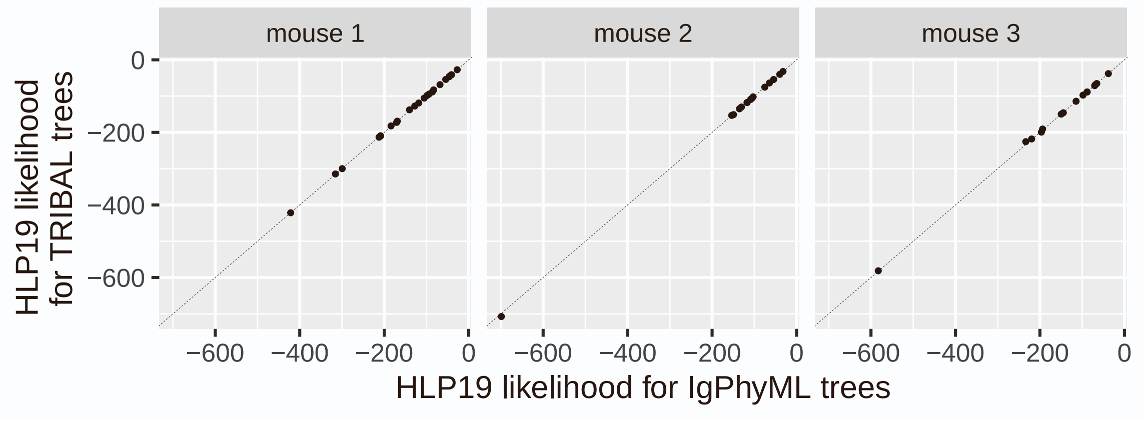

Figure S7. Scatterplot comparing HLP19 likelihood for IgPhyML trees to the HLP19 likelihood computed for TRIBAL trees for ABC datasets, Related to Figure 5.

dataset	clonotypes k	total cells n	median cells per clonotype	max cells per clonotype	median distinct isotypes per clonotype
mouse 1	24	224	7.5	31	2
mouse 2	15	218	7	81	3
mouse 3	15	157	7	39	3

Table S2. Summary of ABC mouse scRNA-seq datasets, Related to Figure 5.

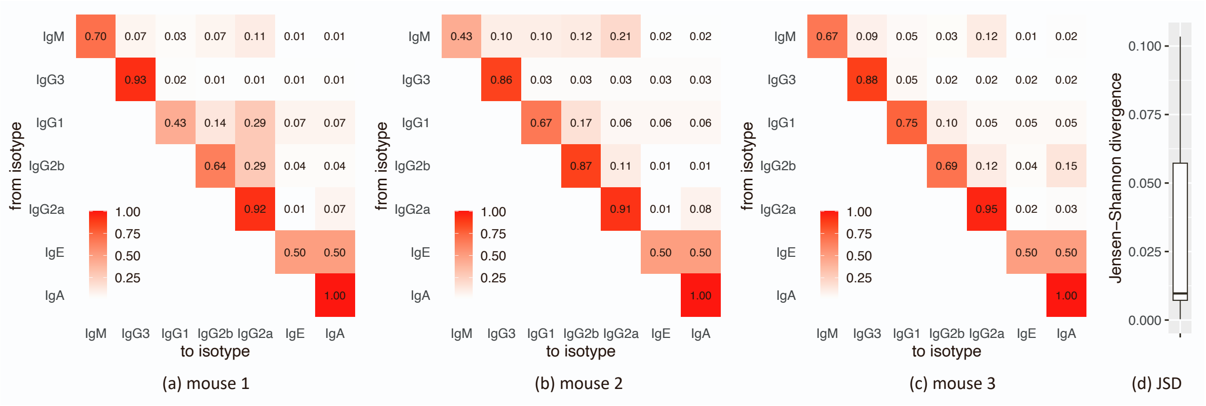

Figure S8. TRIBAL inferred isotype transition probabilities for ABC datasets, Related to Figure 5. (a) Isotype transition probabilities for Mouse 1. (b) Isotype transition probabilities for Mouse 2. (c) Isotype transition probabilities for NP-Mouse 3. (d) The distribution of Jensen-Shannon divergence (JSD) for pairwise comparisons of rows of the inferred isotype transition probability matrices for IgM through Ig2c. IgE was excluded from comparison due to a lack of observed B cells within each dataset to yield informative estimates. IgA is excluded as the inference of this row is trivial.

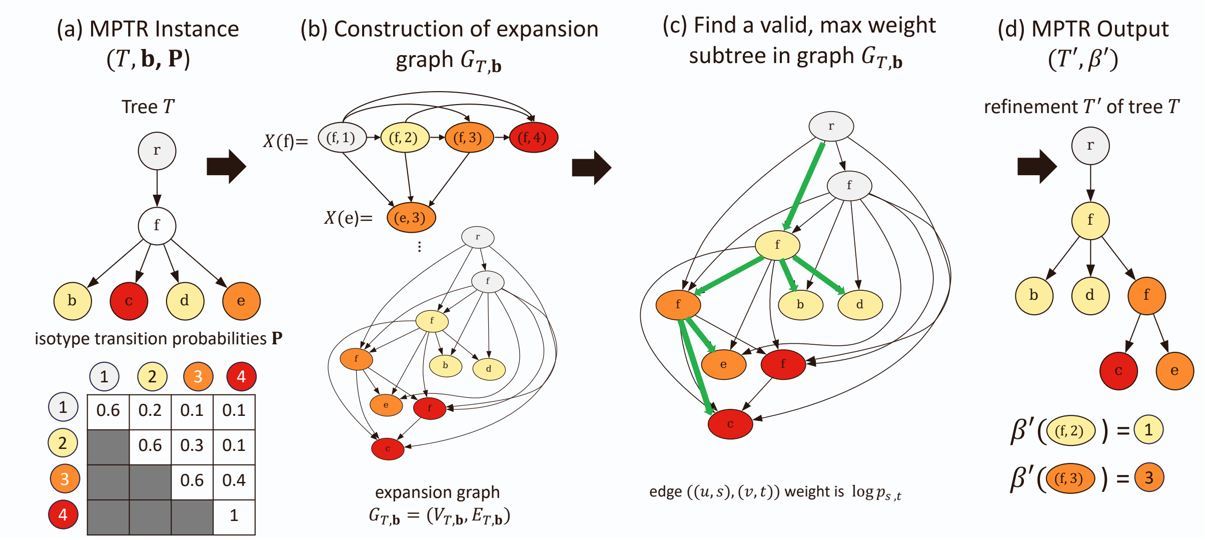

	1	2	3	4
1	0.6	0.2	0.1	0.1
2		0.6	0.3	0.1
3			0.6	0.4
4				1

Figure S9. Algorithm for solving the MPTR problem (a) An instance $(T, \mathbf{b}, \mathbf{P})$ of the MPTR problem. (b) To construct the expansion graph $G_{T,\mathbf{b}}$ for tree T whose leaves have isotypes $\mathbf{b}$, each original node u in $V(T)$ corresponds to a set $X(u)$ of nodes in $G_{T,\mathbf{b}}$. Edges are added to capture all transitory refinements of tree T. (c) We use the expansion graph $G_{T,\mathbf{b}}$ with weighted edges to find a valid, maximum weight subtree in $G_{T,\mathbf{b}}$, depicted in green. (d) This selected subtree is an optimal solution (T', β') to the MPTR problem instance $(T, \mathbf{b}, \mathbf{P})$. Related to Figure 1, 2 and STAR Methods.

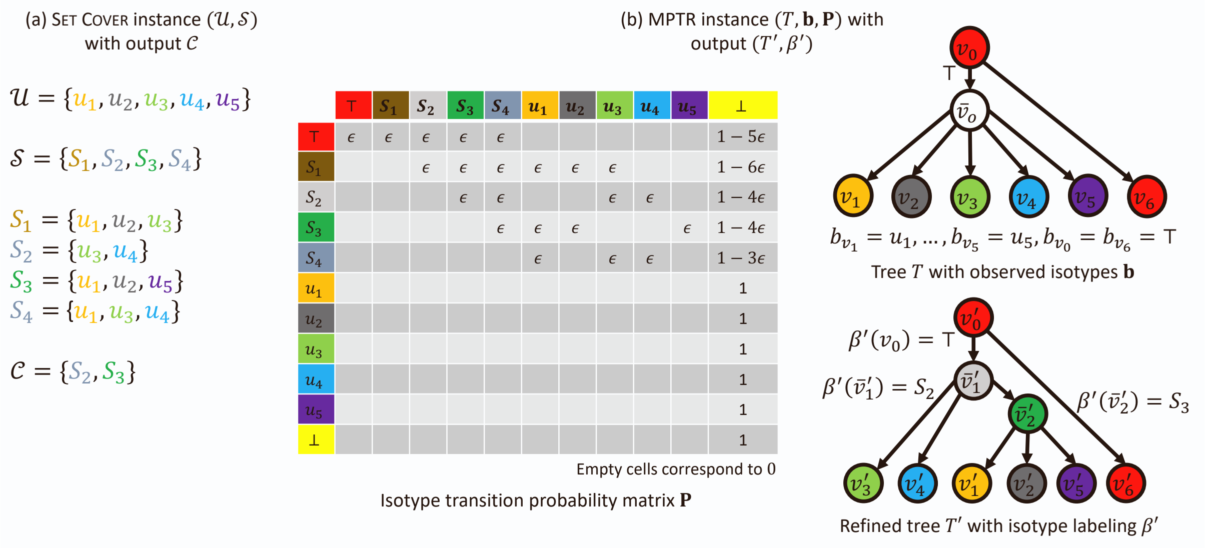

	$\top$	S_1	S_2	S_3	S_4	u_1	u_2	u_3	u_4	u_5	$\bot$
$\top$	ϵ	ϵ	ϵ	ϵ	ϵ						$1-5\epsilon$
S_1			ϵ	ϵ	ϵ	ϵ	ϵ	ϵ			$1-6\epsilon$
S_2				ϵ	ϵ			ϵ	ϵ		$1-4\epsilon$
S_3					ϵ	ϵ	ϵ			ϵ	$1-4\epsilon$
S_4						ϵ		ϵ	ϵ		$1-3\epsilon$
u_1											1
u_2											1
u_3											1
u_4											1
u_5											1
$\bot$											1

Figure S10. Polynomial time reduction from SET COVER to MPTR, Related to STAR Methods. (a) shows a SET COVER instance $(\mathcal{U}, \mathcal{S})$, with the corresponding minimum set cover $\mathcal{C}$. The constructed MPTR instance $(T, \mathbf{b}, \mathbf{P})$, along with the output (T', β') is shown in (b). Isotypes are indicated through colors. The mapping function R is omitted, with the isotypes directly represented by elements, subsets, $\top$, or $\bot$. The empty boxes in the transition probability matrix $\mathbf{P}$ corresponds to 0.

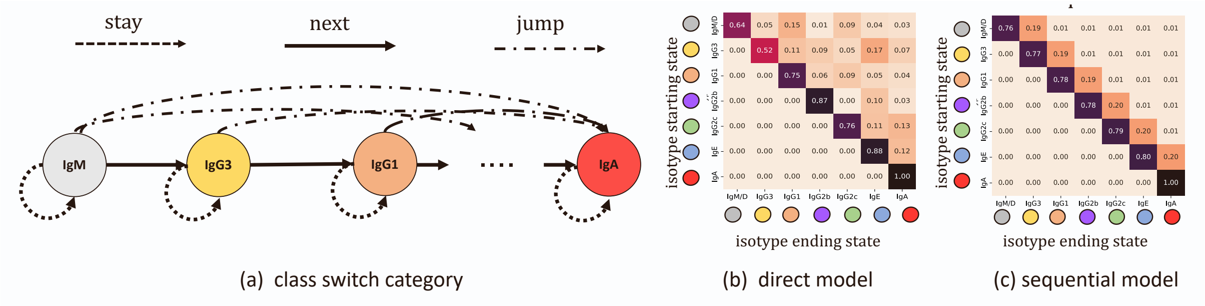

Figure S11. Class switch recombination models for *in silico* experiments, Related to Figure 3. a) Examples of different isotype transition probability parameter groups. (b) Examples of simulated isotype transition probabilities $\mathbf{P}$ for the direct model of CSR. In the direct model, when a B cell class switches is no systematic preference for transition to the *next* sequential state or *jumping* to a non-contiguous isotype. (c) In the sequential model, a B cell undergoing CSR has a strong affinity for the *next* contiguous heavy chain locus.

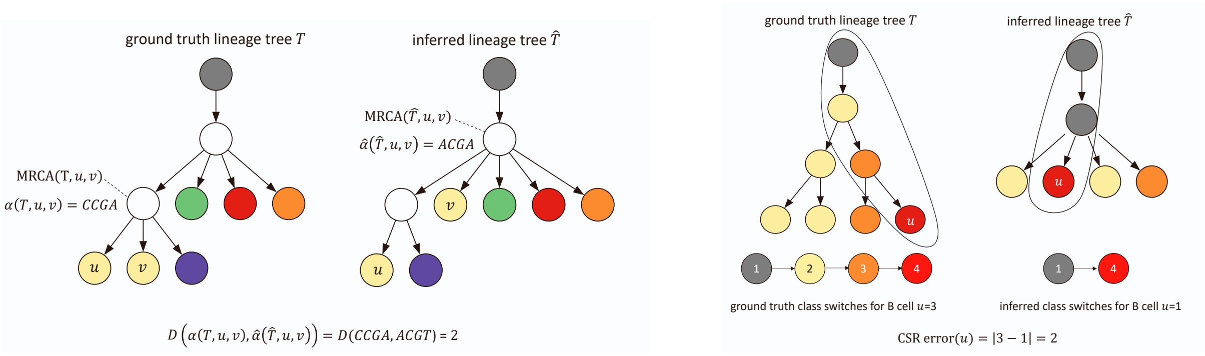

(A) MRCA distance **(B)** CSR error

Figure S12. Performance metrics for B cell lineage tree inference. (a) An example calculation for MRCA distance leaves u and v. (b) An example calculation of CSR error for lineage u, Related to Figure 3.
